# Characterization of wastewater-derived bacteriophages infecting *Enterococcus faecalis* in Bulgaria: insights into the novel phage vB_SEF_8

**DOI:** 10.3389/fmicb.2025.1674800

**Published:** 2025-12-11

**Authors:** Yoana Kizheva, Tsveta Dimova, Maria Pandova, Yoana Gladicheva, Ralitsa Petrova, Tsvetelina Paunova-Krasteva, Zoltan Urshev, Sergei Ivanov, Petya Hristova

**Affiliations:** 1 Department of General and Industrial Microbiology, Faculty of Biology, Sofia University “St. Kliment Ohridski”, Sofia, Bulgaria; 2 Stephan Angeloff Institute of Microbiology, Bulgarian Academy of Science, Sofia, Bulgaria; 3 RnD Department, LB Bulgaricum Plc., Sofia, Bulgaria; 4 Research Group: Microbiological Risks in the Environment, Sofia University “St. Kliment Ohridski”, Sofia, Bulgaria

**Keywords:** antibiotic resistance, *Enterococcus faecalis*, food safety, phage therapy, *Saphexavirus* phages, wastewater

## Abstract

*Enterococcus faecalis* is an opportunistic pathogen associated with nosocomial infections, food spoilage, and reduced efficacy of orally administered medications in patients with Parkinson’s disease. Its genetic adaptability, particularly in acquiring virulence and antibiotic resistance genes, poses a significant challenge in treatment. Тhus, the development of new and effective approaches, such as phage therapy, is crucial in the fight against *E. faecalis*. The main goal of this study was to establish the biological characteristics of three bacteriophages (designated as vB_SEF_8, vB_SEF_13 and vB_SEF_15) isolated from wastewater in Bulgaria and their potential to eliminate *E. faecalis*. The host ranges of the phages were determined primarily using *E. faecalis* strains (*n* = 29), although other species within the genus *Enterococcus* were also included. All three phages targeted only *E. faecalis* strains, including antibiotic-resistant or multidrug-resistant strains. The phages showed broad pH (4.0–10.5) and temperature (up to 80 °C) stability, formed clear plaques, with maximal titers reached at various MOIs. After 9 months at 4 °C, only a slight titer reduction was observed (up to 2 log_10_ PFU/mL). RFLP analysis revealed genetic diversity among the three phage isolates. The phage with the broadest host range (vB_SEF_8) was characterized in more details. TEM observation revealed elongated head and long noncontractile tail. vB_SEF_8 possessed linear dsDNA and lacked genes associated with lysogeny, antibiotic resistance, or virulence. Phylogenetic analysis and the calculated pairwise intergenomic distance showed that vB_SEF_8 is a novel species within the *Saphexavirus* genus, class *Caudoviricetes*. The phage also successfully inhibited *E. faecalis* in a milk-based matrix. The collected data demonstrate that vB_SEF_8 holds significant potential as an antibacterial and therapeutic agent against *E. faecalis* in settings where the presence of this bacterium is undesirable.

## Introduction

The growing prevalence of antibiotic-resistant (ABR) bacteria, resulting from decades of excessive and uncontrolled antibiotic use, is a major problem, worldwide. As the humanity entered the post-antibiotic era, bacteriophages were rediscovered as a promising solution for addressing this challenge through an approach known as phage therapy. Although phage therapy has diverse applications (García et al., 2023; Runa et al., 2021; Holtappels et al., 2021; Kizheva et al., 2023; Shopova et al., 2023; Huang et al., 2022), one of its primary goals is to target bacterial pathogens responsible for severe infections in mammals. These include those associated with hospital-acquired infections (HAIs)—a major global cause of morbidity and mortality—among which enterococci, streptococci, staphylococci, and *Clostridium difficile* are recognized as particularly severe agents (McFee, 2009; Rossolini et al., 2010).

*Enterococcus faecalis* is considered as one of the leading agents in severe HAIs like urinary tract and wound infections, bacteraemia, neonatal sepsis, peritonitis, and endocarditis (Proença et al., 2012). The main challenge in managing *E. faecalis* infections is its antibiotic resistance, driven by the bacterium’s highly adaptable genome and its capacity to acquire and spread resistance and virulence genes via horizontal gene transfer (Rossolini et al., 2010; Hegstad et al., 2010). As a result, there has been a notable increase in *E. faecalis*—related infections in recent years possibly due to the development of resistance to multiple antibiotics (Proença et al., 2012). Moreover, in the human GIT, *E. faecalis* has been associated with another concerning issue, particularly in individuals with Parkinson’s disease (PD). The bacterium produces an enzyme - tyrosine decarboxylase, which converts the orally administrated medicine L-DOPA to dopamine in the GIT, which results in decreased effectiveness of this medicine in such patients (Hong et al., 2024). On the other hand, *E. faecalis* has been frequently found in various food products (dairy products, vegetables, meat, fermented sausages etc.), representing an undesirable microbial contamination with the potential to cause food spoilage (Pandova et al., 2024; del Río et al., 2019).

Considering this, *E. faecalis* can be regarded as a pathogen of critical importance, requiring strong efforts toward the development of alternative, non-antibiotic therapeutic strategies like phage-based solutions. However, effective phage therapy against this pathogen depends on isolation and characterization of diverse phages and exploration of their therapeutic potential. Indeed, phages infecting *E. faecalis* have been previously described, and summarized data indicate a predominance of the genera *Efquatrovirus* and *Saphexavirus*. Although these genera were originally classified within the order Caudovirales, family *Siphoviridae*, both are now placed under the class *Caudoviricetes* (Rodríguez-Lucas and Ladero, 2023; International Committee on Taxonomy of Viruses (ICTV), n.d.). For example, four *E. faecalis* phages have been isolated from sewage and investigated as novel additions to the existing pool of phages targeting *E. faecalis*-associated infections (Di Lallo et al., 2021). In another study, the therapeutic potential of phage vB_EfaS_HEf13 has been demonstrated, with authors concluding that it may serve as a promising agent against *E. faecalis*-related dental infections, particularly recurrent or refractory apical periodontitis (Lee et al., 2019). The potential of phage SFQ1 (vB_EfaS_SFQ1) to disrupt *E. faecalis* biofilms has also been investigated, with results indicating notable efficacy (Song et al., 2023). Several *Siphoviridae* bacteriophages have been isolated from the oral cavities of patients with root canal infections and have demonstrated strong lytic activity against *E. faecalis*, including effective biofilm disruption both *in vitro* and *in vivo*, as shown in a zebrafish infection model (Al-Zubidi et al., 2019). In a similar study, bacteriophage vB_EfaS_PHB08 also demonstrated effective lytic activity and biofilm degradation, with its encoded endolysin, Lys08, showing enhanced antimicrobial performance in the presence of Mn^2+^ ions (Yang et al., 2020). Additionally, beyond whole-phage applications, endolysins derived from lytic phages have also been considered as promising antibacterial agents. For example, endolysin (pEF51) obtained from *E. faecalis* phage PEf771 has shown potential in disrupting *E. faecalis* biofilms and also a broader bactericidal activity compared to the phage itself (Xiang et al., 2024). Interestingly, phages targeting *E. faecalis* have been investigated in a novel context—in eliminating the bacterium from the gastrointestinal tract of patients with PD. In mouse models, phage application reduced *E. faecalis* in the GIT, resulting in improved L-DOPA efficacy (Hong et al., 2024).

To enhance the success of phage therapy, it is essential to maintain a pool of phages targeting the relevant bacterial species. This underscores the need to isolate and evaluate the therapeutic potential of diverse phages to expand the range of available treatment options. In this context, the primary aim of this study was to isolate and establish the biological properties of bacteriophages infecting *E. faecalis*, one of the leading causative agents in HAIs. As a result, three *E. faecalis* bacteriophages were isolated from wastewater. Key phage characteristics were studied revealing phenotypic and molecular diversity among them. The in-depth study of one of these phages (vB_SEF_8) showed that it effectively lysed ABR *E. faecalis* strains and inhibited *E. faecalis* in a milk-based matrix, indicating its potential for use in various settings where this bacterium poses a threat. We believe that our findings contribute to the expanding body of research aimed at establishing bacteriophages’ potential as antibacterial agents. According to the available data, our study represents a novel investigation into the isolation and characterization of potentially therapeutic bacteriophages infecting *E. faecalis* in Bulgaria.

## Materials and methods

### Isolation of *E. faecalis* strains from wastewater

Wastewater samples were collected at the entrance of wastewater treatment facilities (WWTF) near cities of Sofia, Varna and Burgas, Bulgaria for a period of 3 months (February–May). The samples were stored in sterile containers for 24 h in a refrigerator, and were first processed to remove larger debris. Next, they were tenfold diluted in sterile saline. Aliquots of 100 μL of selected dilutions were directly plated on enterococci selective agar medium Slanetz and Bartley (SB) (HiMedia, Mumbai, India) and cultivated at 37 °C for 24–48 h. Single colonies with specific red - brownish color were isolated as pure cultures in deMan, Rogosa and Sharpe (MRS) broth medium (HiMedia, Mumbai, India). The newly isolated strains were Gram stained and MALDY-ToF mass spectrometry (Autobio, Zhengzhou, China) was used for species identification. According to the manufacturer, score values between 9.5 and 10.0 indicate reliable subspecies-level identification and values from 9.0 to 9.5 are considered reliable at the species level. Scores ranging from 6.0 to 9.0 support identification at the genus level, whereas values below 6.0 are regarded as unreliable.

### Antibiotic susceptibility assay of newly isolated *E. faecalis* strains

The newly isolated *E. faecalis* strains were tested for antibiotic susceptibility to 11 antibiotics [Ampicillin (AMP), 2 μg/disc; Imipenem (IPM), 10 μg/disc; Norfloxacin (NX), 10 μg/disc; High-Level Gentamicin (HLG), 30 μg/disc; High-level Streptomycin (HLS), 300 μg/disc; Teicoplanin (TEI), 30 μg/disc; Vancomycin (VA), 5 μg/disc; Eravacycline (ERV), 20 μg/disc; Tigecycline (TGC), 15 μg/disc; Linezolid (LZ), 10 μg/disc; and Nitrofurantoin (NIT), 100 μg/disc] via the Kirby–Bauer disk diffusion method (Bauer et al., 1966). Log bacterial cultures were obtained after cultivation of the strains in MRS agar at 37 °C for 24 h. Bacterial suspensions were prepared in sterile saline (10^8^ CFU/mL, MacFarland 1.3) and plated on Mueller-Hinton agar (MHA, Merck KGaA, Darmstadt, Germany). The antibiotic paper disks were surface placed on Petri dishes and cultivated for 24 h at 37 °C. The interpretation of the results was done according to The European Committee on Antimicrobial Susceptibility Testing (EUCAST Version 15.0, 2025). For the purposes of this study, isolates resistant to two classes of antibiotics were classified as ABR, whereas those resistant to three classes - as multi-drug resistant (MDR).

### Bacteriophage isolation and purification

A wastewater sample, collected as described above, was first processed to remove larger debris. Then the sample was filtered through a 0.22 μm pore size membrane filter to remove the residual bacterial debris (Corning Incorporated). Six *E. faecalis* strains were used as initial host strains (Table 1; Pandova et al., 2024). Phage isolation and purification were done via double agar overlay plaque assay (DAOPA) (Kropinski et al., 2009). The host strains were cultivated on MRS agar for 24 h at 37 °C until obtaining log bacterial cultures. One hundred μL cell suspensions (10^8^ CFU/mL), prepared in sterile saline, were mixed with 100 μL filtered wastewater sample, 30 μL 1 M CaCl_2_ and 3 mL soft MRS agar (0.45% agar content) and poured onto the solid MRS agar plates, supplemented with CaCl_2_ to a final concentration 10 mM (MRS-Ca). Plates were incubated at 37 °C for 24 h and observed for plaques formation. Single plaques were picked with sterile plastic needle and purified via three consecutive cultivations with the respective host strain in MRS-Ca broth.

**Table 1:** Bacterial strains used as initial host for bacteriophage isolation and host range analyses.

Number	Test-microorganisms	Species identification, score value**	Reference	Phage activity		
				vB_SEF_8	vB_SEF_15	vB_SEF_13
1.	*E. faecalis* ATCC 29212	ATCC		−	+	+
2.	*E. faecalis* WeS3 ABR	9.653	This study	+	−	−
3.	*E. faecalis* WeS4	9.528	This study	−	−	−
4.	*E. faecalis* WeS10 MDR	9.648	This study	−	−	+
5.	*E. faecalis* WeS11	9.508	This study	−	−	−
6.	*E. faecalis* WeS13	9.058	This study	−	−	−
7.	*E. faecalis* WeS14	9.339	This study	−	−	−
8.	*E. faecalis* WeB2	9.457	This study	+	−	−
9.	*E. faecalis* WeB5	9.670	This study	−	−	−
10.	*E. faecalis* WeB8 ABR	9.692	This study	−	−	−
11.	*E. faecalis* WeB11	9.232	This study	+	−	−
12.	*E. faecalis* WeV1	9.628	This study	−	−	+
13.	*E. faecalis* WeV11 ABR	9.646	This study	−	−	−
14.	*E. faecalis* WeV17	9.543	This study	+	−	−
15.	*E. faecalis* BM2*	Pandova et al. (2024)		+	−	−
16.	*E. faecalis* BM3			+	−	−
17.	*E. faecalis* BM4			+	−	−
18.	*E. faecalis* BM5			+	−	−
19.	*E. faecalis* BM6			+	−	−
20.	*E. faecalis* BM7*			+	−	−
21.	*E. faecalis* BM8*			+	−	−
22.	*E. faecalis* BM9			+	−	−
23.	*E. faecalis* BM12			-	−	+
24.	*E. faecalis* BM13*			−	−	+
25.	*E. faecalis* BM14			−	−	−
26.	*E. faecalis* BM15* ABR			−	+	−
27.	*E. faecalis* CM4			−	−	−
28.	*E. faecalis* YFC1 ABR			−	−	+
29.	*E. faecalis* YFC3*			−	−	−
30.	*E. faecium* ATCC 19434	ATCC		−	−	−
31.	*E. faecium* CM1	Pandova et al. (2024)		−	−	−
32.	*E. faecium* BY14			−	−	−
33.	*E. faecium* BY16			−	−	−
34.	*E. faecium* MFC2			−	−	−
35.	*E. faecium* DK1			−	−	−
36.	*E. durans* CM2			−	−	−
37.	*E. durans* YFC4			−	−	−
38.	*E. mundtii* CA1			−	−	−
39.	*E. mundtii* CA8			−	−	−
40.	*E. casseliflavus* CA2			−	−	−
41.	*E. gilvus* CA3			−	−	−
42.	*E. pallens* CA10			−	−	−
43.	*E. malodoratus* CA11			−	−	−
44.	*E. coli* ATCC 8739	ATCC		−	−	−
45.	*S. aureus* ATCC *6538*			−	−	−

Bold*, strains used as hosts in the initial isolation of the phages from wastewater; Score value**, species identification based on Maldi ToF MS score values; ABR, strains, resistant to two classes of antibiotics; MDR, multi-drug resistant strains; ATCC, American type culture collection.

### Determination of host range of the phages

For host range determination 45 bacterial strains, belonging to 10 bacterial species, were used (Table 1). Spot testing assay (STA) was applied (Kutter, 2009). All bacterial strains were cultivated overnight on appropriate agar media - MRS for enterococci, Tryptic Soy Agar (TSA, Merck KGaA, Darmstadt, Germany) for *Escherichia coli* ATCC 8739 and *Staphylococcus aureus* ATCC 6538. Bacterial suspensions were prepared in sterile saline (10^8^ CFU/mL, MacFarland 1.3). Aliquots of 100 μL of each suspension were mixed with 30 μL 1 M CaCl_2_ and 3 mL melted soft agar (MRS, TSA) and poured on the respective solid agar medium (MRS-Ca/TSA-Ca). Phage crude lysates were prepared in MRS-Ca broth. They were 10-fold diluted up to 10^−9^ in sterile saline and aliquots of 10 μL of each dilution were spot inoculated on the solid agar plates. Double agar plates were cultivated at 37 °C for 24 h and the appearance of plaques of bacterial lysis was considered as positive outcome.

### Determination of the morphology of phage plaques formed on *E. faecalis* lawn

All newly isolated phages were cultivated with their respective bacterial host via DAOPA described above. The morphology of the plaques was determined on the bases of observation of resulted plaques after cultivation (clear or turbid and presence/absence of halo). Plaque dimensions were measured with electronic caliper and at least three separate plaques for each phage were studied. The final results were expressed as mean ± SD.

### Determination of optimal multiplicity of infection of the phages

The best phage:bacteria ratio, resulting in highest phage titers, i.e., optimal multiplicity of infection (MOI) was studied for all newly isolated phages. Phages and their respective bacterial host were cultivated in 10 mL MRS-Ca broth in different ratios for the different MOI values 0.01, 0.1, 1, 10, and 100. The optimal MOI was established after measuring of phage titers after cultivation at 37 °C for 4 h. The highest phage titers indicated best phage:bacteria ratio, i.e., optimal MOI for phage propagation.

### pH and thermal stability of the phages

The phage thermal stability and tolerance to different pH were studied according Park et al. (2023). Phage buffer containing 10mMTris—HCl, 10 mM MgSO_4_, 68 mM NaCl and 1 mM CaCl_2_ was prepared in sterile dH_2_O and pH was corrected (2.0; 4.0; 5.92; 7.5; 9.0; 10.5; 13.0) (Msimbira et al., 2016). Phage lysates with known titers were mixed with pH solutions in ratio 100 μL:900 μL and incubated at 37 °C for 3 h after which the phage titers were counted via STA. The thermal stability of the phages was established after measuring of the number of the viable phage particles before and after the incubation of crude phage lysates at different temperatures (28°С, 37°С, 50°С, 65°С, 80°С, and 95°С). The duration of incubation was 2 h. The experiments were done in triplicates and the final results were expressed as mean ± SD.

### Influence of storage temperature on phages’ viability

The phage isolates were cultivated with their respective host bacterial strain at the optimal MOI in 10 mL MRS-Ca broth and stored at 4 °C for 9 months. The initial titers of the resulted phage lysates were measured via STA and compared to the phage titers after storage.

### Extraction of phages’ genetic material

Crude phage lysates were prepared in 30 mL MRS-Ca broth and initial titers were determined by STA; only lysates with titers ≥10^8^ PFU/mL were selected for further analysis. Half volume of precipitation solution (30% PEG8000, 3 M NaCl) was added to one volume of lysate. The mixtures were incubated overnight at 4 °C and then centrifuged at 10,000×*g* for 30 min in a refrigerated centrifuge. Supernatants were discarded, and the resulting pellets were resuspended in 500 μL TE buffer (10 mM Tris–HCl, 1 mM EDTA, pH 8.0). Subsequently, 10 μL DNase (1 U/μL) and 10 μL RNase (40 mg/mL) were introduced, followed by incubation at 37 °C for 1 h. Then the samples were supplemented with 40 μL of 0.5 M EDTA (final concentration 40 mM) followed by incubation at 65 °C for 10 min for DNase inactivation. Next, 500 μL Lysis buffer GB from a commercial DNA extraction kit (AccuPrepR Genomic DNA Extraction Kit, Bioneer, Republic of Korea) and 20 μL of Proteinase K (20 mg/mL) were added and samples were incubated for 1 h at 65 °C. Samples were mixed with 1 mL absolute ethanol and applied to the columns provided in the same kit with subsequent steps performed according to the protocol of kit manufacturer.

### Restriction fragment length polymorphism (RFLP) assay on newly isolated phages

RFLP approach was applied to determine the genetic diversity between the newly isolated phages. Phage DNAs were digested with *Hind*III (Thermo Fisher Scientific). The reaction mixtures were prepared according manufacturer’s recommendations and incubated at 37 °C for 6 h. The resulted RFLP patterns were visualized electrophoretically in 1.5% agarose gel at 100 V for 1 h. *λ*-Hind III digest was used as molecular weight marker (TaKaRa Bio, Europe).

### Virion morphology of phage vB_SEF_8

The virion morphology of selected phage isolate was established via transmission electron microscopy (TEM). The preparation of the samples for TEM analyses were prepared according procedure described before (Kizheva et al., 2023). The observations were carried out using a transmission electron microscope JEOL JEM 2100 operating at 200 kV (JEOL Ltd., Tokyo, Japan).

### Sequencing and bioinformatics of the genome of vB_SEF_8

The whole genome of phage vB_SEF_8 was sequenced in MicrobesNG, Birmingham, UK. The company’s hybrid service includes combination of two sequencing approaches—Oxford Nanopore Technologies (R10.4.1 Flowcells) and 2 × 250 bp Illumina short read. Raw reads were evaluated, processed and assembled by Microbes NG. The assembled genome was analyzed and annotated via CLC sequence viewer (Version 7.6) and Pharokka software (Galaxy Version 1.3.2.), respectively (Bouras et al., 2022). ABRicate tool (Galaxy version 1.0.1.) was used for prediction of antibiotic resistance and virulence associated genes (Seemann, 2016). tRNAscan was used for finding tRNA coding sequences (Lowe and Eddy, 1997). Comparative genome alignment and its corresponding graphical representation were performed using EasyFig (Sullivan et al., 2011).

### Phylogenetic analyses

The phylogenetic analysis, based on whole genome nucleotide sequences, was carried out by the VICTOR web service,^1^ a method for the genome-based phylogeny and classification of prokaryotic viruses (Meier-Kolthoff and Göker, 2017). All pairwise comparisons of the nucleotide sequences were conducted using the Genome-BLAST Distance Phylogeny (GBDP) method under settings recommended for prokaryotic viruses (Meier-Kolthoff et al., 2013; Meier-Kolthoff and Göker, 2017). Whole genomes of 21 *Enterococcus* phages (*Saphexavirus*, *n* = 18, *Efquatrovirus*, *n* = 3) and 1 *Streptococcus* phage (*Saphexavirus*) were obtained from GenBank and used in phylogenetic analyses. The phylogenetic distance between vB_SEF_8 and the other known *E. faecalis* phages was calculated with VIRIDIC (Virus Intergenomic Distance Calculator) and the results were interpreted according the criteria given from International Committee on Taxonomy of Viruses (ICTV) (Moraru et al., 2020).

### vB_SEF_8 potential as antibacterial agent in milk—based matrix

We studied the potential of phage vB_SEF_8 to suppress the growth of *E. faecalis* in simulated milk—based matrix. Tryptic Soy broth + 2% skim milk (TSB-SM) was used as a cultivation medium and was chosen as being the most similar to milk food matrix. *E. faecalis* strain BM8 was used as host strain. Overnight bacterial culture grown on MRS agar was used for preparation of bacterial suspension (10^8^ CFU/mL) in sterile saline as described above. Pure phage lysate was prepared in MRS-Ca broth as described above and phage titer was measured via DAOPA (10^9^ PFU/mL). The experiment was conducted as follows: control 1 (C1)—mono cultivation of *E. faecalis* strain BM8 in 10 mL TSB-SM; control 2 (C2)—mono cultivation of phage vB_SEF_8 in 10 mL TSB-SM; test sample (TS)—co-cultivation of *E. faecalis* BM8 and vB_SEF_8 in 10 mL TSB-SM in MOI 0.01. The duration of the experiment was 4 h at 37 °C. The number of viable bacterial cells (in C1 and in the TS) and the viable phage particles (in C2 and in the TS) were measured via Plate count method on SB agar medium and STA on MRS agar medium with *E. faecalis* BM8 as host strain, respectively. The experiment was conducted in four independent replicates.

### Statistical analysis

The data were analyzed by Microsoft Excel v. 2508 using XLMiner Analysis ToolPak with ANOVA single factor or two-factor with replication. A *p*-value below 0.05 was considered statistically significant.

## Results

### Antibiotic susceptibility testing of the newly isolated *E. faecalis* strains

For the purposes of this study 13 bacterial strains, with presumed belonging to *E. faecalis*, were isolated from wastewater. All isolates were Gram-positive cocci and were subsequently identified as *E. faecalis* via MALDI-ToF (Table 1). Their phenotypic antibiotic susceptibility is presented in Supplementary Table S1. The results revealed that all isolates were susceptible to six of the 11 tested antibiotics, while all strains were resistant to ampicillin. All strains were found “susceptible, increased exposure” (I) to imipenem, except strain WeS10 which was resistant. Three strains (WeS3, WeB8 and WeV11) were resistance to two groups of antimicrobials—ampicillin, high-level streptomycin and/or high-level gentamycin, and considered in this study as ABR. One of the newly isolated strains (WeS10) was resistant to three groups - ampicillin, imipenem and norfloxacin. As these antibiotics are agents from three antimicrobial categories it was categorized as MDR strain (Magiorakos et al., 2012).

### Bacteriophage isolation and host range determination

**Figure 1:**
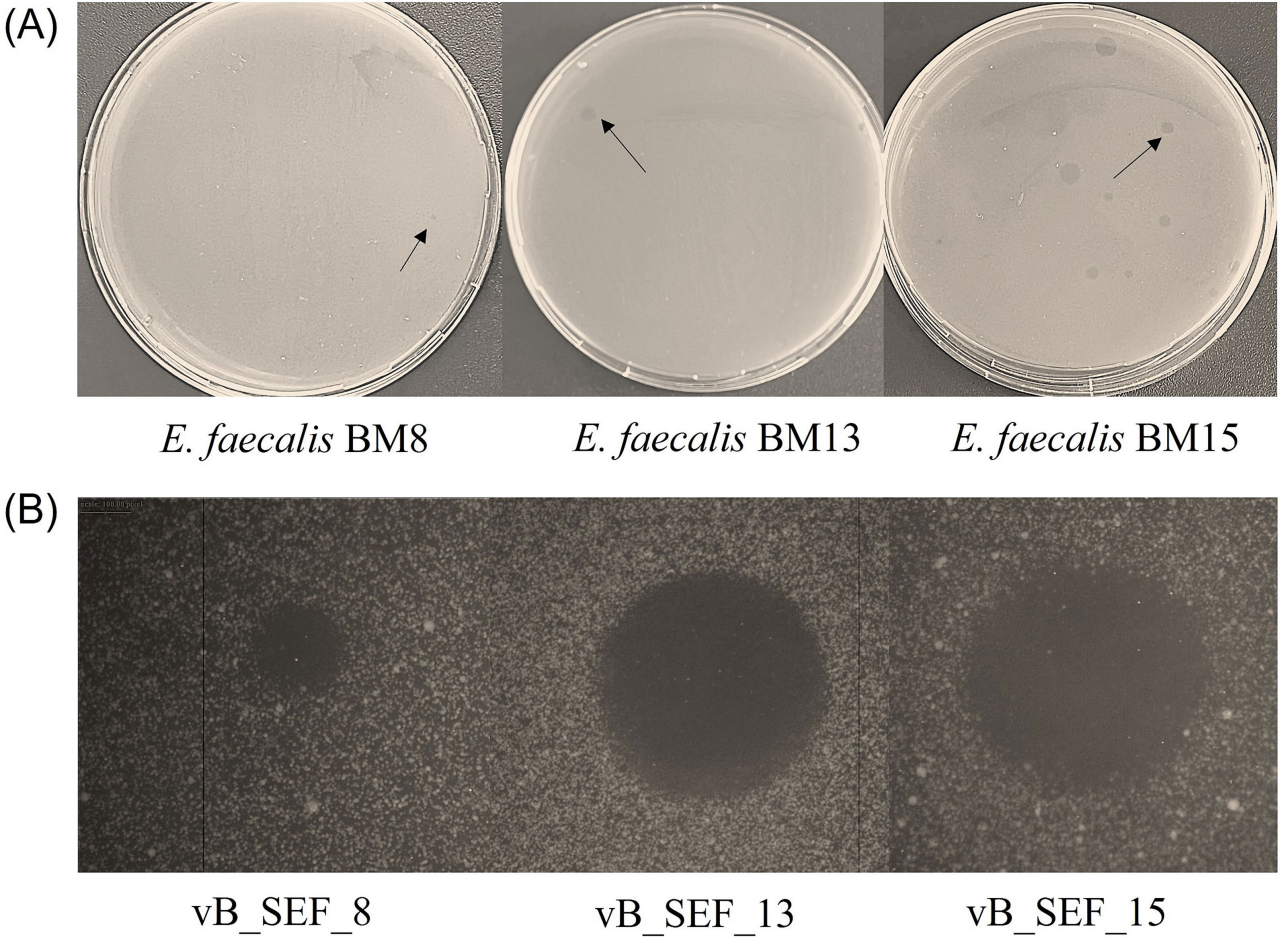
**(A)** Plaques of bacterial lysis observed on MRS-Ca agar plates after cultivation of the wastewater with bacterial hosts. Three *E. faecalis* strains (BM8, BM13, and BM15) were used as initial hosts for isolation of bacteriophages. The black arrows indicate the exact plaques from which the phages were isolated. The smallest plaque (diameter 0.68 mm) was observed on the *E. faecalis* BM8 lawn, from which the novel phage vB_SEF_8 was isolated. **(B)** Clear plaques obtained via DAOPA after cultivation of the newly isolated phages with their initial respective hosts. The plaque diameters were measured by electronic caliper and at least four independent measurements for each phage were conducted. The final values were obtained as mean ± SD: vB_SEF_8 (*d* = 0.84 ± 0.119 mm), vB_SEF_13 (*d* = 3.38 ± 0.483 mm) and vB_SEF_15 (*d* = 2.51 ± 0.336 mm).

Six *E. faecalis* strains (five from human origin and one from young feta cheese) were used as initial hosts for phage isolation (Table 1). After cultivation of the host strains with the wastewater sample, plaques from bacterial lysis were observed only on the three of the strains of human origin (*E. faecalis* BM8, *E. faecalis* BM13 and *E. faecalis* BM15). Notably, only a single plaque appeared on the *E. faecalis* BM8 and BM13 lawns, whereas multiple plaques formed on the BM15 lawn, one of which was selected for further study (Figure 1A). As a result, three *E. faecalis* specific bacteriophages, designated as vB_SEF_8, vB_SEF_13 and vB_SEF_15, were isolated with vB_SEF_8 isolated from the smallest plaque (0.68 mm), and vB_SEF_13 from the largest (3.21 mm). The host range determination revealed that all phages were capable to infect different number but only strains of the species *E. faecalis*. No lytic activity was observed against the remaining eight enterococcal species, including *E. faecium* (Table 1). Each phage isolate was capable to infect different *E. faecalis* strains, which suggests that the newly isolated phages differed from each other. Moreover, we established that the MDR strain *E. faecalis* WeS10 and the ABR strain *E. faecalis* YFC1 were lysed by phage isolate vB_SEF_13. Additionally, two ABR strains WeS3 and BM15 were susceptible to vB_SEF_8 and vB_SEF_15, respectively. Generally, 66% (*n* = 19) of the tested *E. faecalis* strains were susceptible to at least one of the newly isolated phages and 34% (*n* = 10) were resistant (seven isolated from wastewater, one with human origin, one from cow milk and one from young feta cheese). Strain *E. faecalis* ATCC 29212 was susceptible to two of the tested phages (Table 1). Phage vB_SEF_8 had the broadest host range as it was capable to lyse 12 out of 29 tested *E. faecalis* strains (41%). Generally, *E. faecalis* strains isolated from wastewater showed lower sensitivity to the newly isolated phages compared to isolates with human origin.

### Determination of plaques’ morphology

The morphology of the plaques produced by the phages during cultivation with their target bacterium is an essential phage characteristic. The newly isolated phages formed clear plaques on *E. faecalis* lawn. The difference was observed only in plaques’ diameters (*d*). As expected, phage vB_SEF_8 formed the smallest plaques (*d* = 0.84 ± 0.119 mm), followed by phage vB_SEF_15 (*d* = 2.51 ± 0.336 mm), while phage vB_SEF_13 generated the largest plaques (*d* = 3.38 ± 0.483 mm) (Figure 1B).

### Determination of optimal multiplicity of infection of the phages

In the MOI analyses conducted in this study we established that phage: bacteria ratio for obtaining of high-titers phage lysates was different for the three phage isolates. For phage vB_SEF_8 this ratio was 0.01, for phage isolate vB_SEF_13–0.1 and for vB_SEF_15–1.

### Establishing of stability of the phages

**Figure 2:**
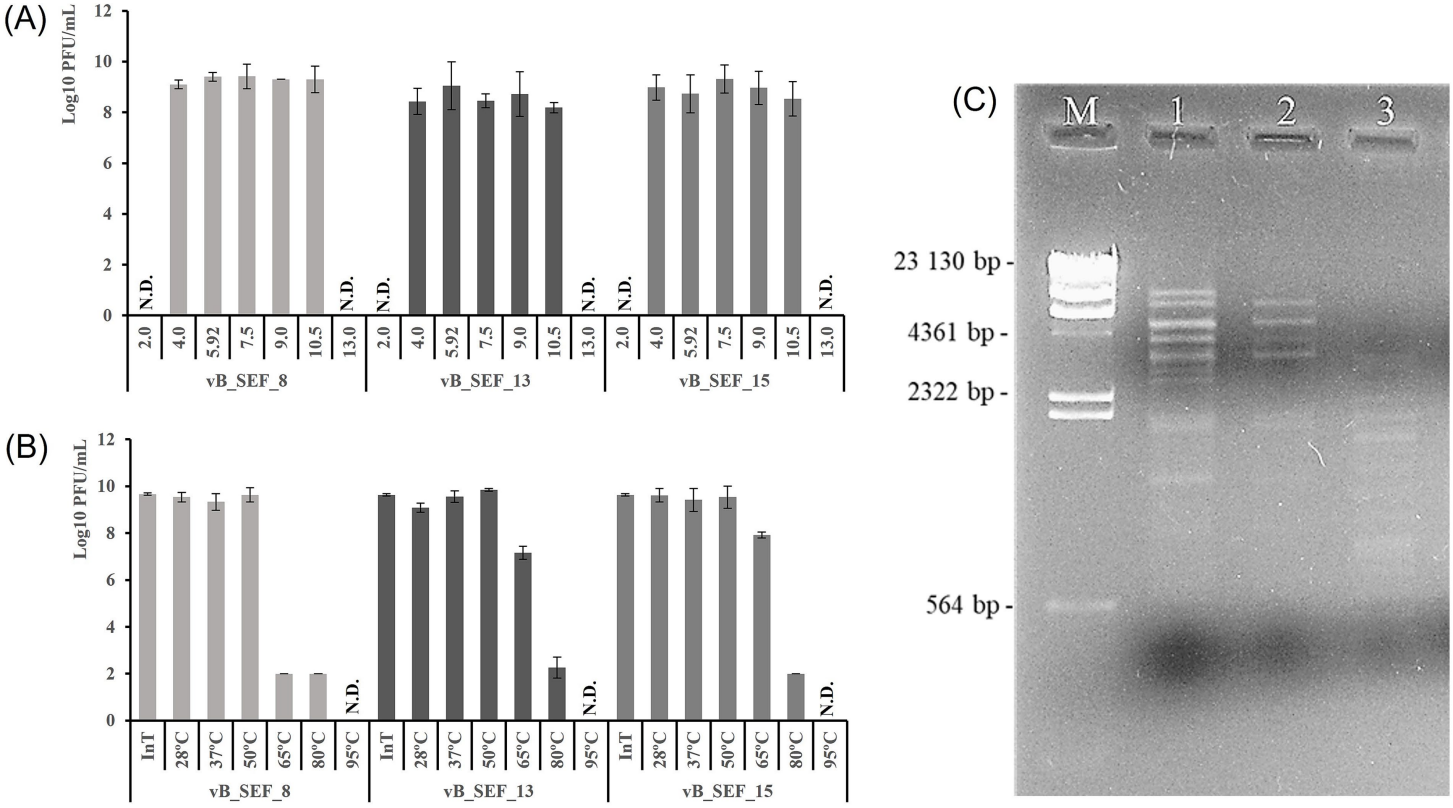
**(A)** Phage titers determined after incubation of the phages in pH buffers with different pH. **(B)** Phage titers determined after incubation of the crude phage lysates at different temperatures (T°C). InT, initial phage titers; N.D., not detected. All values are expressed as means ± SD of three independent biological replicates. **(C)** Agarose gel showing the restriction patterns of the newly isolated phages obtained after digestion of phage DNAs with *Hind*III. 1—phage vB_SEF_13, 2—phage vB_SEF_15, and 3—phage vB_SEF_8. M—*λ*-*Hind*III digest used as molecular weight marker (TaKaRa Bio, Europe).

Generally, all phages completely lost their lytic activity after incubation in extremely acidic and extremely basic conditions (pH 2.0 and 13.0) and remained viable in wide pH range (between 4.0 and 10.5). The ANOVA analysis showed no significant differences in the titers of individual phages, suggesting that pH values between 4.0 and 10.5 did not negatively affect phage activity (*p* = 0.514). However, statistical comparison between the phages revealed significant differences (*p* = 0.0043), with phage vB_SEF_8 demonstrating the greatest stability, reflected by the highest titers across the tested pH range (Figure 2A). Notably, no statistically significant differences were established on phage susceptibility to temperature up to 50 °C (*p* = 0.189) i.e., phage titers remained high. All phages were completely disactivated after incubation at 95 °C. Interestingly, incubation at 65 °C demonstrated temperature-dependent phage activity, with differential heat tolerance among the phages resulting in highly significant differences in their titers (*p* < 0.05). The most temperature sensitive phage isolate was vB_SEF_8 as its titer decreased dramatically (>7 log_10_ PFU/mL) after incubation at 65 °C while the two other isolates were more stable at this temperature (titers decrease up to 3 log_10_ PFU/mL). However, viable phage particles of the three newly isolated phages were detected even after incubation at 80 °C, although at very low titers (Figure 2B). The impact of storage conditions on phages’ lytic activity was determined by counting their titers after storage of phage crude lysates at 4 °C for 9 months. The most stable isolate was vB_SEF_15 as its titer decreased only 1 log_10_ PFU/mL, followed by the two other isolates—titer decrease up to 2 log_10_ PFU/mL.

### Genetic diversity between phage isolates established by RFLP analyses

The genetic material of all phage isolates was extracted and subjected to digestion with restriction enzyme *Hind*III. The successful restriction reaction and the obtained results showed that the newly isolated phages possess double-stranded DNA (dsDNA). Moreover, genetic diversity between phage isolates was revealed as they formed different RFLP patterns (Figure 2C).

### Virion morphology of phage vB_SEF_8

**Figure 3:**
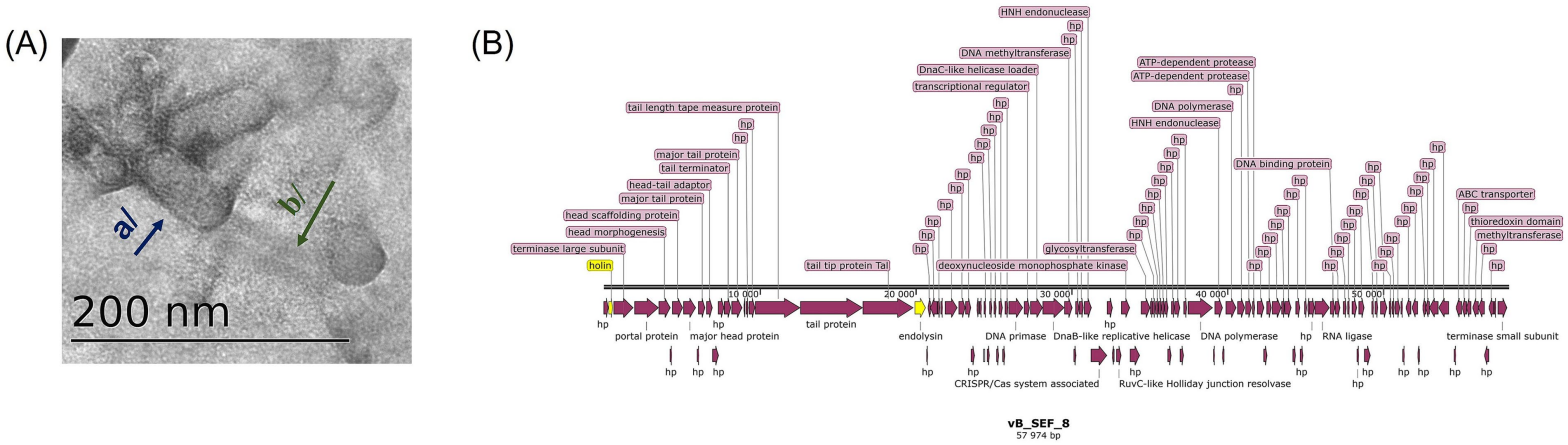
Morphological and genomic characterization of phage vB_SEF_8. **(A)** TEM micrograph of the virion particles of phage vB_SEF_8. Fresh phage suspension (10^9^ PFU/mL) was prepared in phage buffer and 50 μL were used to prepare negatively stained (2% uranyl acetate was used), formvar-coated grids for examination by TEM. The blue arrow (a) indicates the elongated capsid of the phage and the green arrow (b)—long noncontractile tail. The dimensions of the virion particles were obtained after at least three independent measurements and are expressed as mean ± SD. The capsids had length of 80 ± 5.07 and width 34.85 ± 3.03. The tail—approximately 150 nm. **(B)** Genome organization of the linear dsDNA of the phage vB_SEF_8. The complete genome of the phage was sequenced with a combination of two sequencing approaches—Oxford Nanopore Technologies (R10.4.1 Flowcells) and 2 × 250 bp Illumina short read. The processed sequence was deposited to the GenBank with accession number PV948781. The annotation of the CDS was made with Pharokka software (Galaxy Version 1.3.2.) and the image was generated with SnapGene (Version 8.1.1.) The yellow arrows indicate the phage encoded holin and endolysin. HP, hypothetical protein.

Phage vB_SEF_8 was chosen for in-depth analyses based mostly on the results obtained from the host range analyses. This phage demonstrated the broadest host range. The TEM observation revealed a tailed phage. The capsid had elongated shape with length 80 ± 5.07 and width 34.85 ± 3.03. The tail was long (approximately 150 nm in length) and noncontractile (Figure 3A). The observed micromorphology of the phage vB_SEF_8 correspond to the those of siphovirus (class *Caudoviricetes*).

### Genome features of phage vB_SEF_8 and phylogenetic relationships

**Figure 4:**
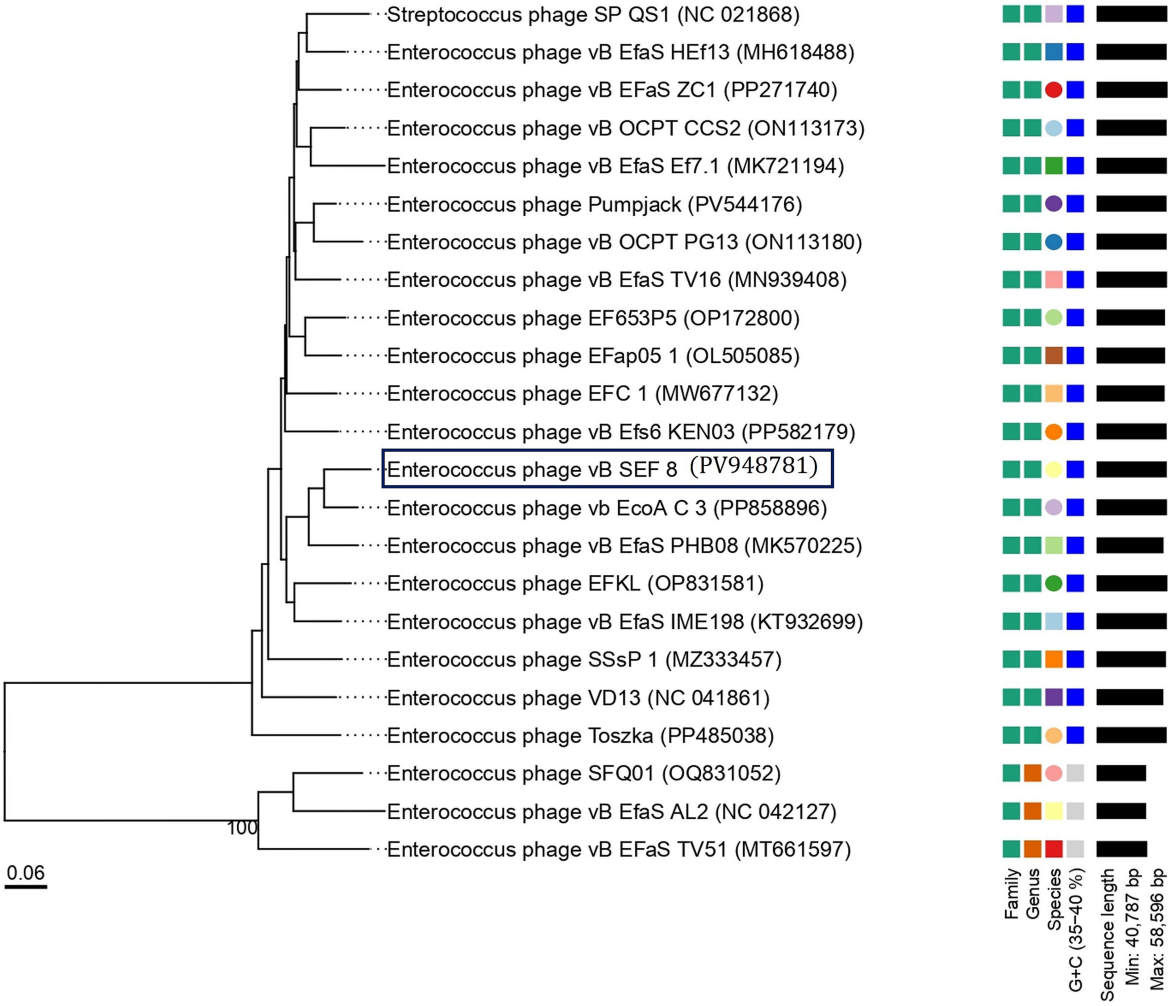
Phylogenetic tree generated based on the whole genome sequences similarity between vB_SEF_8 (dark blue rectangle) and selected *Enterococcus* phages, members of genera *Saphexavirus* and *Efquatrovirus*. The newly isolated phage clustered together with its closest relatives - *Enterococcus* phage vB EfaS PHB08 (MK570225) and *Enterococcus* phage vB_EcoA C-3 (PP858896), both members of genus *Saphexavirus*. The image was generated by VICTOR web service (https://ggdc.dsmz.de/victor.php), accessed July, 2025.

**Figure 5:**
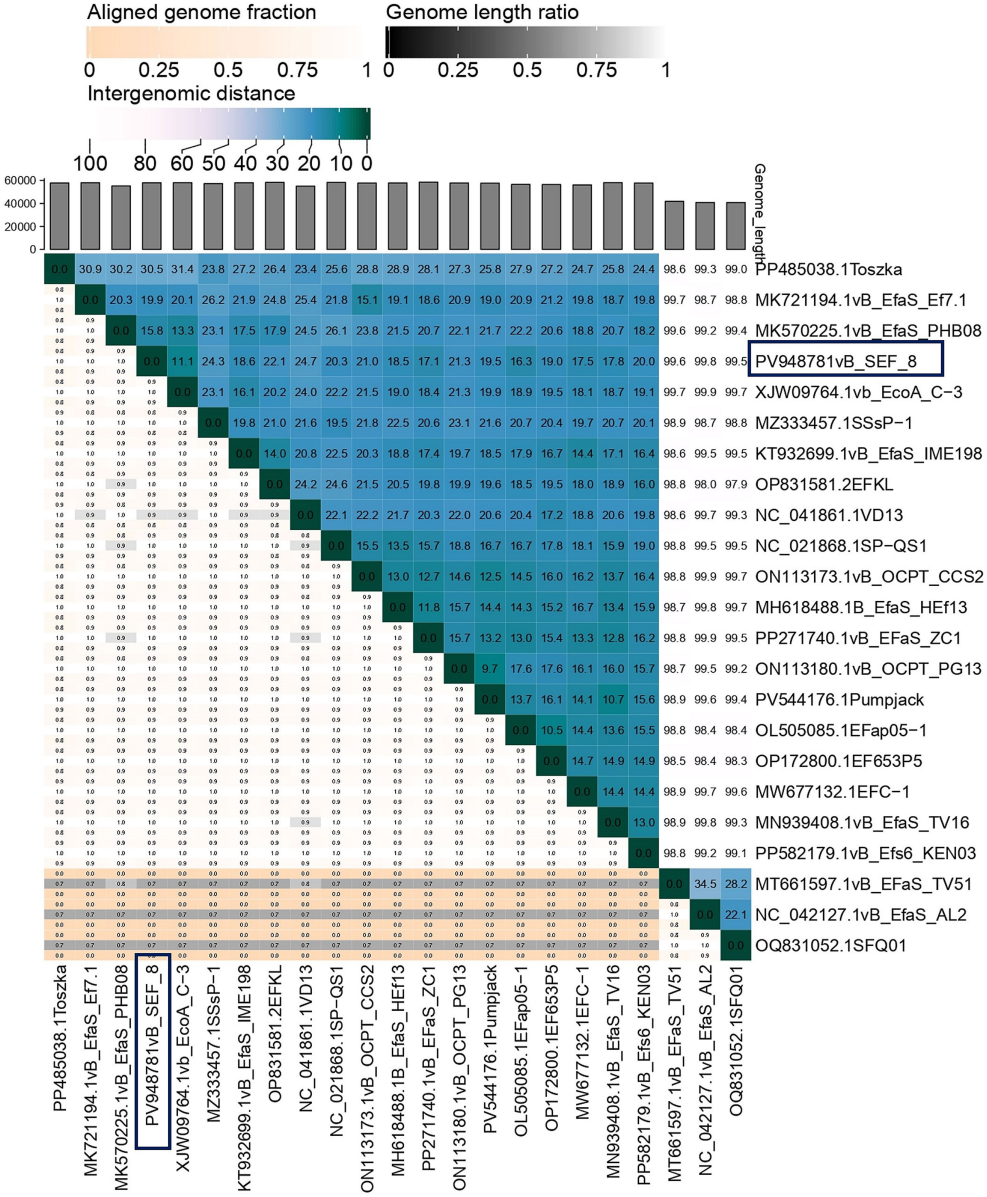
Heatmap showing the calculated pairwise intergenomic distance between vB_SEF_8 (dark blue rectangles) and the *Enterococcus* phages, members of genera *Saphexavirus* and *Efquatrovirus*, used in the analyses. The distance between vB_SEF_8 and its closest relatives was between 11.1 and 15.8% and thus the similarity was calculated to 88.9 and 84.2%, respectively. According to the ICTV species and genus demarcation thresholds for newly isolated viruses (95 and 70%, respectively) vB_SEF_8 was considered novel species within genus *Saphexavirus.* The image was generated by VIRIDIC (Virus Intergenomic Distance Calculator), accessed on July, 2025.

**Figure 6:**
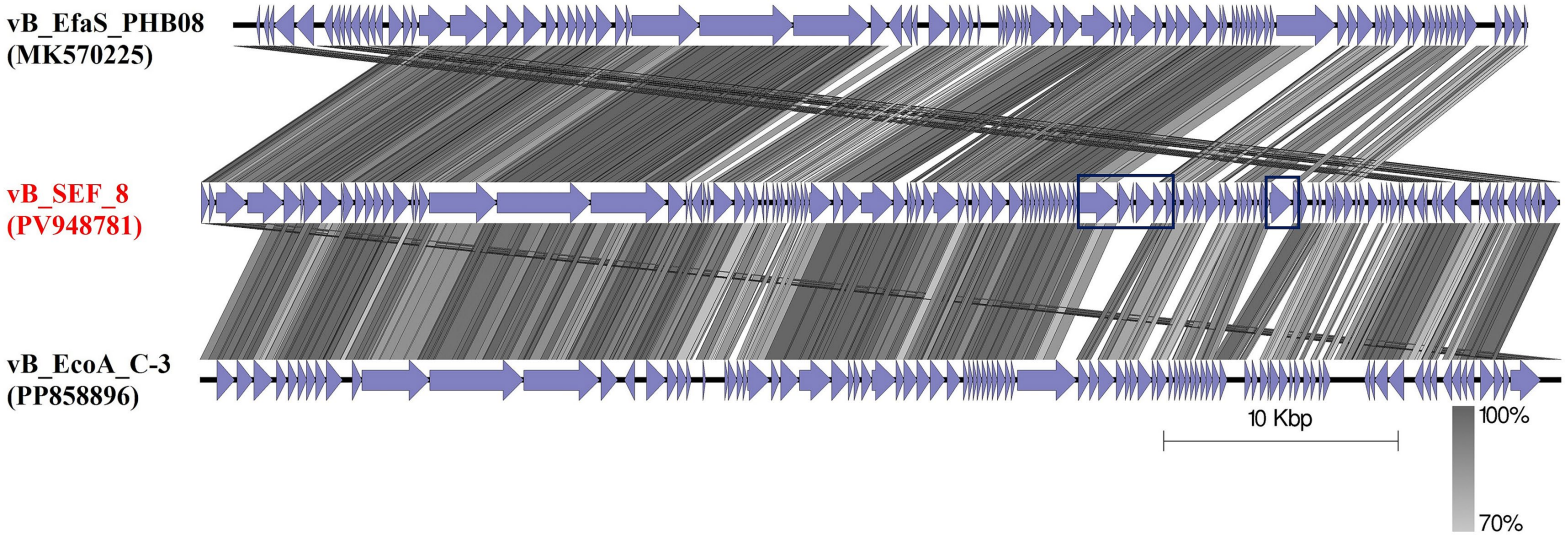
Comparative genome alignment of phage vB_SEF_8 and its closest relatives (vB EfaS PHB08 and vB_EcoA C-3). The image was generated with EasyFig program. The purple arrows indicate the predicted CDS in the genomes of the three phages. The genetic similarity profiles of vB_SEF_8 and the two other phages are showed as percent homology (grayscale). The blue rectangles indicates the major differences in the genome of vB_SEF_8 compared to the two other genomes.

The complete genome of phage vB_SEF_8 was sequenced and deposited at the GenBank under the accession number PV948781. Phage vB_SEF_8 was found to possesses a linear dsDNA with 57,974 bp length and 39.9% G + C content (Figure 3B). The genome contains 75 predicted open reading frames (ORFs), out of which 55 (73%) on the positive strand, and 20 (27%) on the negative. Of the 122 coding sequences (CDS) identified in the genome, 30% were successfully annotated using Pharokka software and found to be responsible for structural proteins, DNA replication, packaging, regulation, and host cell lysis (holin and endolysin) (Table 2). The remaining 70% were identified as hypothetical proteins. No genes encoding putative integrases as well as acquired genes for antibiotic resistance and/or virulence were detected. Two tRNA coding sequences were found with tRNAscan—SE program (Trp (CCA) and Arg). The constructed phylogenetic tree grouped the phage with related members of the genus *Saphexavirus*, within the class *Caudoviricetes* (formerly the family *Siphoviridae*). The results showed that the closest relatives to the phage were *Enterococcus* phage vB EfaS PHB08 (MK570225) and *Enterococcus* phage vB_EcoA C-3 (PP858896) (Figure 4). The calculated pairwise intergenomic distance showed a significant phylogenetic distance between vB_SEF_8 and vB EfaS PHB08–15.8% and, vB_EcoA C-3–11.1% (Figure 5). The ICTV defines species and genus demarcation thresholds for newly isolated viruses as 95 and 70% nucleotide sequence identity, respectively. Based on these criteria and the calculated similarity percentages between these two phages and vB_SEF_8 (84.2 and 88.9%, respectively) we can conclude that vB_SEF_8 is a novel species within genus *Saphexavirus* (Moraru et al., 2020). The conducted comparative genome analysis revealed that vB_SEF_8 possess similar genome arrangement to its closest relatives (Figure 6). Notably, some genetic differences were observed, mainly in the genes encoding DNA polymerase, RNA ligase, and HNH endonuclease. An insertion consisting of four CDSs was identified that was absent in the two other closely related genomes.

**Table 2:** Annotated CDS with predicted function in the genome of vB_SEF_8.

Number	CDS	Position, bp	Strand	Product
1.	002	336–584	+	holin
2.	003	647–1,918	+	terminase large subunit
3.	004	1,975–3,510	+	portal protein
4.	005	3,522–4,277	+	head morphogenesis
5.	007	4,388–5,062	+	head scaffolding protein
6.	008	5,111–5,917	+	major head protein
7.	010	6,073–6,513	+	major tail protein
8.	011	6,573–6,977	+	head-tail adaptor
9.	014	7,748–8,182	+	tail terminator
10.	015	8,203–8,892	+	major tail protein
11.	019	9,724–12,609	+	tail length tape measure protein
12.	020	12,623–16,615	+	tail protein
13.	021	16,627–19,854	+	tail tip protein Tal
14.	022	19,929–20,642	+	endolysin
15.	038	25,193–25,339	+	host RecBCD nuclease inhibitor
16.	042	25,956–26,900	+	DNA primase
17.	043	26,975–27,328	+	transcriptional regulator
18.	044	27,377–28,153	+	DnaC-like helicase loader
19.	045	28,165–29,529	+	DnaB-like replicative helicase
20.	046	29,542–30,066	+	DNA methyltransferase
21.	050	30,829–31,269	+	HNH endonuclease
22.	051	31,262–32,290	+	CRISPR/Cas system associated
23.	054	32,885–33,190	+	RuvC-like Holliday junction resolvase
24.	055	33,187–33,756	+	deoxynucleoside monophosphate kinase
25.	057	34,473–35,036	+	glycosyltransferase
26.	067	36,961–37,188	+	transcriptional regulator
27.	069	37,466–39,064	+	DNA polymerase
28.	071	39,163–39,675	+	HNH endonuclease
29.	073	39,897–40,565	+	DNA polymerase
30.	075	41,173–41,562	+	ATP-dependent protease
31.	076	41,584–41,772	+	ATP-dependent protease
32.	089	45,593–46,555	+	RNA ligase
33.	090	46,602–46,907	+	DNA binding protein
34.	114	54,635–55,006	−	ABC transporter
35.	116	55,330–55,590	−	thioredoxin domain
36.	117	55,708–56,088	−	methyltransferase
37.	122	57,334–57,933	+	terminase small subunit

### Influence of phage vB_SEF_8 on *E. faecalis* in milk-based matrix

**Figure 7:**
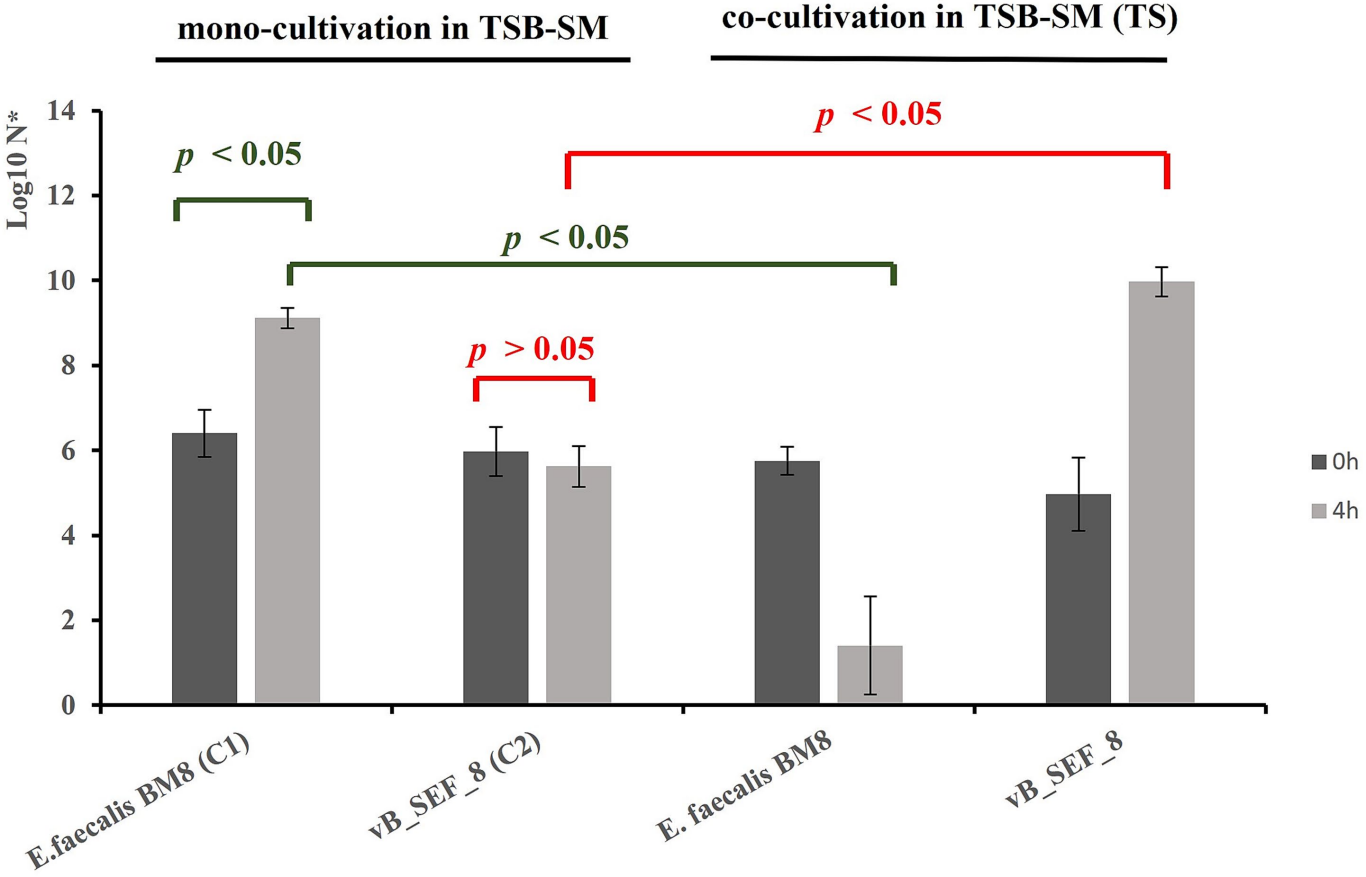
Potential of phage vB_SEF_8 to suppress the growth of *E. faecalis* BM8 in simulated milk conditions for 4 h. All values are presented as means ± SD from four independent trials. The red marks indicate the comparison of vB_SEF_8 titers in C2 and TS, and the dark green marks—bacterial cell count in C1 and TS. The *p*-value below 0.05 was considered statistically significant. N*—CFU/mL (bacteria) or PFU/mL (phage).

This experiment aimed to determine the inhibitory effect of phage vB_SEF_8 on *E. faecalis* BM8 growth in simulated milk—based matrix with presumption of its potential application as antibacterial agent in food. At the beginning of the experiment the viable cell count of *E. faecalis* BM8 cells and viable phage particles of vB_SEF_8 was determined in both the controls (C1 and C2) and in the TS. As it can be seen on Figure 7 an interaction between the bacteria and the phage occurred in the TS as statistically significant decrease in bacterial cell number in TS compared to the C1 (*p* < 0.05) was established at the fourth hour of the experiment. Simultaneously, a significant statistical increase in phage titer was detected in TS compared to C2 (*p* < 0.05). These results showed that vB_SEF_8 has the potential to suppress the growth of *E. faecalis* in milk—based matrix. In parallel, in C1 the bacterial cell number increased statistically significant at the fourth hour of the experiment (*p* < 0.05) while the number of viable phage particles in C2 was stable during the entire experiment (*p* = 0.0585). This demonstrated that the medium itself did not influence the viability of the phage.

## Discussion

The development of phage therapy, as one of the most promising approach in the combat with MDR bacteria, relies on the joint efforts in the isolation and characterization of diverse phages infecting target harmful bacteria. As one of the leading agents in HAI, *E. faecalis* is considered a pathogen of key importance and isolation of potentially therapeutic phages against it is of critical importance. Moreover, the bacterium’s role in food product spoilage and its influence on drug efficacy in PD patients highlight additional areas where phage therapy may offer significant benefits (McAuley et al., 2012; Hong et al., 2024).

In this paper we report the characterization of three *E. faecalis*—specific bacteriophages isolated in Bulgaria. Wastewater was selected as source for phage isolation due to its composition from domestic, industrial, and clinical effluents, where the persistence of *E. faecalis* and its phages was expected. Generally, wastewater and sewage are among the most frequently mentioned sources of isolation of phages infecting *E. faecalis* (Ji et al., 2023; Pazhouhnia et al., 2022; Al-Zubidi et al., 2019; Lee et al., 2019; Song et al., 2023; Di Lallo et al., 2021).

The newly isolated phages differed in their host range as they demonstrated different strain specificity. Phage vB_SEF_8 showed the broadest host range. The species specificity of the phages was confirmed as they were capable of infecting only *E. faecalis* strains. Our results are in compliance with previously reported data concerning the host range of *E. faecalis*-specific bacteriophages (Al-Zubidi et al., 2019; Song et al., 2023). Generally, 66% of tested *E. faecalis* strains in our study were susceptible to at least one of newly isolated phages. Additionally, the ability of the studied phages to target ABR and MDR strains of *E. faecalis* suggests their potential for application as therapeutic agents. Over the past decades, antibiotic resistance in *E. faecalis*-associated infections has emerged as a major concern (Yu et al., 2020). In this regard the interest toward phages, capable to destroy MDR bacteria rose significantly (Keen, 2012). Therefore, it is not surprising that studies similar to ours aim precisely to isolate phages that specifically infect MDR *E. faecalis* (Ali et al., 2025). Nevertheless, 10 of the tested *E. faecalis* strains in our study (34%) were found to be resistant to the newly isolated phages. Bacterial resistance to phage infection relies on diverse mechanisms and the formation of capsule plays a key role in it (Labrie et al., 2010). In our previous study, whole genome sequencing revealed that some of the *E. faecalis* strains used in the following study (e.g., CM4) carried the complete *cps* operon suggesting their ability to form capsule (Thurlow et al., 2009; Pandova et al., 2024). Other strains, like BM5, BM12, and BM15 carried only separate genes, which suggested blocked capsule formation ability. However, in this study the latter three strains were found to be phage susceptible, while *E. faecalis* CM4—phage resistant. Thus, the role of the capsule in the phage infection of our *E. faecalis* strains needs further clarification. In another study, the role of the enterococcal polysaccharide antigen (EPA) as a receptor for phage adsorption, thereby mediating strain-specific infection, has been investigated (Al-Zubidi et al., 2019).

Each newly isolated bacteriophage, intended for phage therapy, must meet several key criteria with the obligately lytic cycle being one of the most essential traits (Hatfull et al., 2022). The morphology of the plaques is the first indirect indication of phages’ replication cycles. All studied phages in our investigation formed clear plaques, which varied only in their dimensions. Such variation suggests that the phages may have different size of the capsid as an inverse relationship was suggested between capsid size and plaque diameter, where smaller plaque size corresponded to larger capsid dimensions (Jurczak-Kurek et al., 2016). Generally, clear plaques are typically indicative of a lytic phage life cycle, while turbid plaques are often associated with lysogeny (Jurczak-Kurek et al., 2016). Similar to our study, other *E. faecalis* bacteriophages have been also reported to form clear plaques, with identical plaque diameters, and subsequently they were found lack genes encoding lysogeny (Song et al., 2023; Lee et al., 2019; Pazhouhnia et al., 2022; Al-Zubidi et al., 2019).

The stability of phage particles in different unfavorable conditions (extreme pH and T°C) as well as after long term storage in nonspecific laboratory conditions (4 °C) are considered desirable phage properties (Hatfull et al., 2022). The phages in our study exhibited robust tolerance across a wide pH range (4.0–10.5) and remained viable after 9 months of cold storage as crude lysates. Their pH stability was similar to those reported before for other *E. faecalis* specific bacteriophages (Lee et al., 2019; Pazhouhnia et al., 2022). However, ensuring the survival of orally administered phages through the harsh stomach environment (pH 1.5–2.0) remains a key challenge, driving ongoing research into protective encapsulation strategies (Yang et al., 2023). In most of the reported cases *E. faecalis* phages lose their activity after incubation at 50°/60 °C and, in rare cases at 70 °C (Yang et al., 2020; Lee et al., 2019; Pazhouhnia et al., 2022). Indeed, previous reports have documented *E. faecalis* siphophages with greater thermal stability, remaining viable at 75 °C for 1 h with titers close to 10^7^ PFU/mL (Ali et al., 2025). In our study, viable phage particles were detected after incubation at 80 °C, albeit in greatly diminished numbers, suggesting a thermal sensitivity that, to some degree, differs from what has been reported to date. Notably, *E. faecalis* is a highly resilient bacterium capable of thriving in extreme environments (pH 11.0 and temperatures above 45 °C) (McHugh et al., 2004; Foulquie Moreno et al., 2006). Due to their comparable or superior stability, phages presented in this study may be highly effective in environments where *E. faecalis* must be strictly controlled, such as hospitals.

The genetic diversity between the newly isolated phages was revealed via RFLP analyses, similar to other reported data (Al-Zubidi et al., 2019; Hallewell et al., 2014; Golomidova et al., 2007).

This, along with the observed differences in host range analyses, plaque morphology, temperature and pH stability assays, and MOI analyses we could hypothesize that the newly isolated phages differed from each other. Based on this, one of the newly isolated phages (vB_SEF_8) was recognized as the most promising isolate for detailed characterization—TEM observation and genome sequencing.

The phage was selected primarily due to its ability to lyse the greatest number of the tested *E. faecalis* strains and moreover, its ability to target ABR *E. faecalis* strain (WeS3) isolated from wastewater. The observed virion morphology revealed the presence of long noncontractile tail typical for siphoviruses. The phage possessed elongated head, similar to other, previously reported *E. faecalis* phages classified within genus *Saphexavirus*, for example: vB_EfaS_TV16, vB_EfaS_HEf13, vB_EfaS_PHB08 and G21-7 (Di Lallo et al., 2021; Lee et al., 2019; Yang et al., 2020; Wang et al., 2024). Interestingly, phage vB_EcoA_C-3, one of the closest relatives to vB_SEF_8 based on phylogenetic analysis, had different capsid morphology while the other closest relative, vB_EfaS_PHB08 had virion morphology similar to that of vB_SEF_8 (Wang et al., 2024; Yang et al., 2020).

The type and the organization of genome of vB_SEF_8 (linear dsDNA, 57,974 bp) were found to be similar to the other reported *Saphexavirus* phages [vB_EfaS_TV16 (MN939408), vB_EfaS_HEf13 (MH618488), vB_EfaS_PHB08 (MK570225), vB_EcoA C-3 (PP858896)]. However, comparative bioinformatic analyses using complete genome sequences of previously reported *E. faecalis* phages available in the GenBank revealed variability in the number of ORFs, with vB_SEF_8 differing from its relatives (vB_EfaS_PHB08–73 ORFs, vB_EfaS_HEf13–72, vB_EfaS_TV16–81, and vB_EcoA_C-3—77). Additionally, a genomic region absent in the genomes of the closest relatives was also observed. In this region we found four CDS (two for hypothetical proteins, and one for DNA polymerase and HNH endonuclease). These findings were not surprising, since phylogenetic analysis categorized vB_SEF_8 as a novel species within the genus *Saphexavirus*. The presence of genes encoding endolysin (CDS 022) and holin (CDS 002) as well as the absence of genes for putative phage integrase suggested the obligately lytic life cycle of vB_SEF_8. This result supports our earlier hypothesis that vB_SEF_8 is an obligately lytic phage, as indicated by its plaque morphology.

Ready-to-eat dairy products (yoghurt, cheese) as well as raw milks used for their production are frequent source of isolation of *E. faecalis* (Morandi et al., 2005; Pandova et al., 2024; Gołas-Pradzynska et al., 2022). These bacteria possess significant temperature tolerance thus they could survive after pasteurization of the raw milk in diary production (McAuley et al., 2012). Thus, their manifestation in milk post-pasteurization may cause food spoilage (Semedo et al., 2003). Additionally, *E. faecalis* has strong ability to acquire and transfer genes for antibiotic resistance and virulence so its presence in food is undesirable (Stępień-Pyśniak et al., 2021). In this regard, the application of bacteriophages in treating temperature resistant *E. faecalis* in milk could be considered possible solution to this problem. In our experiment we demonstrated the potential of the phage vB_SEF_8 to significantly suppress the growth of *E. faecalis* cells in milk-based matrix for 4 h. The phage’s ability to withstand high temperatures (up to 80 °C) makes it a promising candidate for use in raw milk as an antibacterial agent, both post- and intra-pasteurization. In a similar experiment, phage Ef-N13 was shown to inhibit *E. faecalis* growth in raw milk, with maximal reduction also observed at the fourth hour of incubation (Ji et al., 2023).

In conclusion, in this study we reported the isolation of diverse phages, targeting ABR and MDR *E. faecalis* strains. One of these phages, vB_SEF_8 was characterized in details and classified as a novel species within the genus *Saphexavirus*, class *Caudoviricetes*. It possesses some of the desired features of therapeutic phages—ability to lyse diverse strains of *E. faecalis*, obligately lytic replication cycle, lack of antibiotic resistance and virulence related genes, good stability in common storage temperatures, good tolerance to wide pH range and high temperatures. Moreover, phage vB_SEF_8 has the potential to be applied as antibacterial agent in diary industry. Our study may be regarded as innovative for Bulgaria and, on a global scale, as a contribution to the existing knowledge in the field of phage therapy against *E. faecalis.* It could also serve as a solid basis for further investigation, particularly in the area of phage resistance mechanisms to phage infection. A major limitation of this study is that the potential of these phages to be applied against severe bacterial pathogens remains confined to the laboratory. Global will is needed to advance phage research beyond the lab and to translate these findings into clinical applications for patients.

## Data Availability

The datasets presented in this study can be found in online repositories. The names of the repository/repositories and accession number(s) can be found in the article/Supplementary material.

## Footnotes

1
https://ggdc.dsmz.de/victor.php
