# Supplementary material for: Characterization of wastewater-derived bacteriophages infecting Enterococcus faecalis in Bulgaria: insights into the novel phage vB_SEF_8
Source: Front Microbiol. 2025 Dec 11;16:1674800. doi: 10.3389/fmicb.2025.1674800 (PMC12739550; doi:10.3389/fmicb.2025.1674800)
Supplement: Supplementary file 1 [file Table_1.docx]

Supplementary Material

**Supplementary Table S1** Phenotypic antibiotic susceptibility of the *E. faecalis* strains isolated from wastewater in this study.

| ***E. faecalis* strains** | **ESCAST interpretation** | | | | | | | | | | |
| --- | --- | --- | --- | --- | --- | --- | --- | --- | --- | --- | --- |
|  | A**MP** | **IPM** | **NX** | **HLG** | **HLS** | **TEI** | **VA** | **ERV** | **TGC** | **LZ** | **NIT** |
| WeS3 | R | I | S | S | R | S | S | S | S | S | S |
| WeS4 | R | I | S | S | S | S | S | S | S | S | S |
| WeS10 | R | R | R | S | S | S | S | S | S | S | S |
| WeS11 | R | I | S | S | S | S | S | S | S | S | S |
| WeS13 | R | I | S | S | S | S | S | S | S | S | S |
| WeS 14 | R | I | S | S | S | S | S | S | S | S | S |
| WeB 2 | R | I | S | S | S | S | S | S | S | S | S |
| WeB 5 | R | I | S | S | S | S | S | S | S | S | S |
| WeB 8 | R | I | S | R | S | S | S | S | S | S | S |
| WeB 11 | R | I | S | S | S | S | S | S | S | S | S |
| WeV 1 | R | I | S | S | S | S | S | S | S | S | S |
| WeV 11 | R | I | S | S | R | S | S | S | S | S | S |
| WeV 17 | R | I | S | S | S | S | S | S | S | S | S |

R – resistant, I - susceptible, increased exposure, S – susceptible.
